# Supplementary material for: Improved Formazan Dissolution for Bacterial MTT Assay
Source: Microbiol Spectr. 2021 Dec 22;9(3):e01637-21. doi: 10.1128/spectrum.01637-21 (PMC8694201; doi:10.1128/spectrum.01637-21)
Supplement: SUPPLEMENTAL FILE 1 — Supplemental material. Download SPECTRUM01637-21_Supp_1_seq5.pdf, PDF file, 0.4 MB [file spectrum01637-21_supp_1_seq5.pdf]

Supplementary material

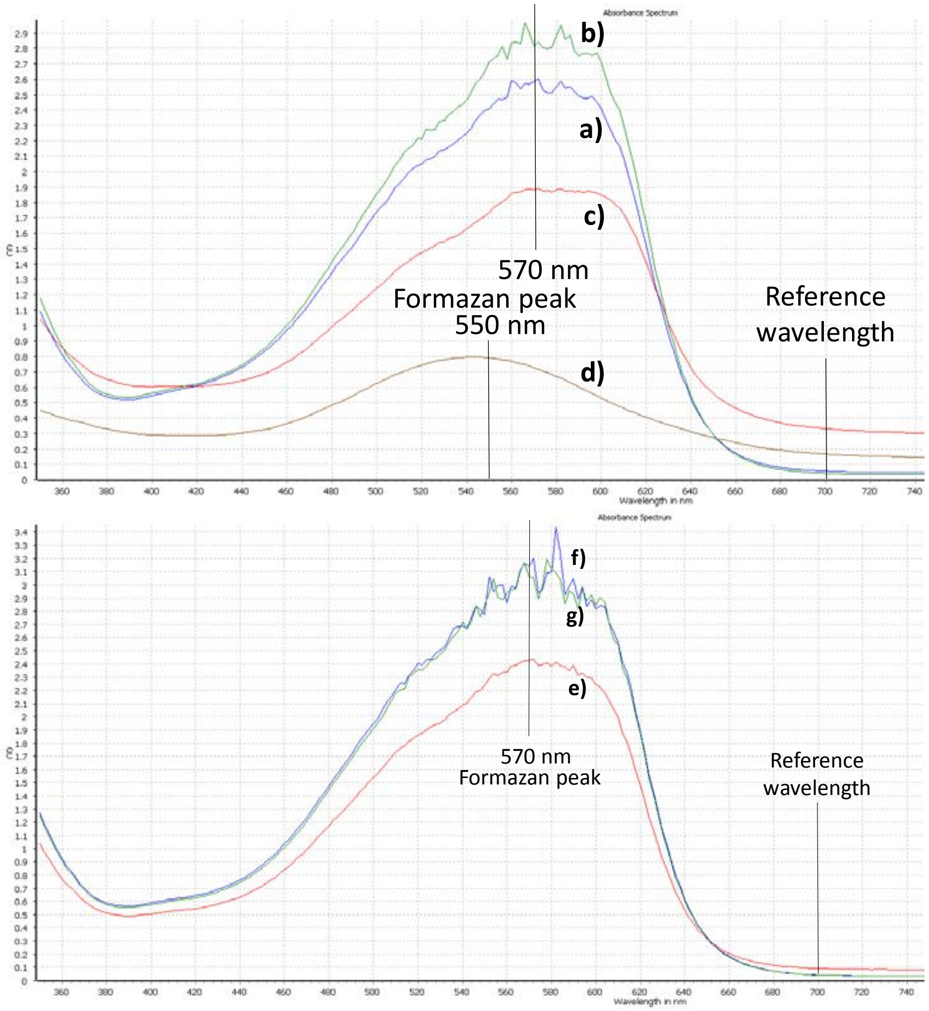

Suppl. Fig. 1. Absorption spectra of pure formazan dissolved in different solvents
Pure formazan crystals were weighed and dispersed in phosphate-buffered saline. Aliquots were serially diluted under constant stirring, and 100 µl portions were added to triplicate wells in a 96-well plate, followed by the addition of 100 µl of the tested solvent. Plates were kept on a shaker at 200 rpm. Spectra were recorded 60 minutes after adding the solvents.
a) 10% SDS-buffered DMF; b) 5% SDS-buffered DMF; c) SDS-EDTA; d) ammonia-DMSO; e) 5% SDS-buffered DMSO; f) 10% SDS-buffered DMSO; g) 15% SDS-buffered DMSO

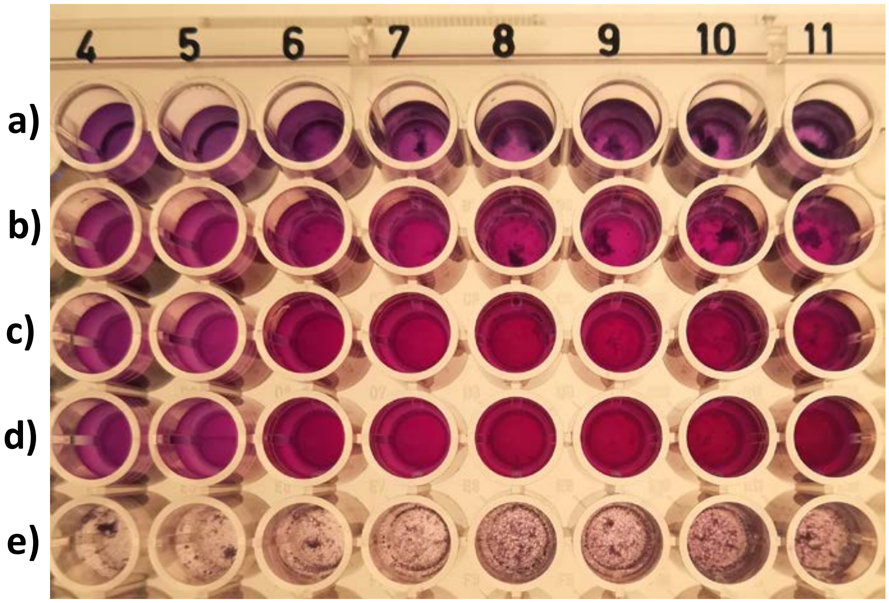

Suppl. Fig. S2. Image of a 96-well plate taken 60 minutes after the addition of the solvents
Pure formazan was added at the quantities indicated below to a duplicate wells containing 100 µl of PBS. Hundred µl of selected solvents were added and the plate was shaken at 200 rpm.
Solvents: a) SDS-EDTA; b) 5% SDS- buffered DMF; c) 7.5% SDS-buffered DMF; d) 10% SDS-buffered DMF; e) ammonia-DMSO
Quantity of formazan (µg per well): wells 4 & 5, **12.5 µg**; wells 6 & 7, **25.0 µg**; wells 8 & 9, **37.5 µg**; wells 10 & 11, **50.0 µg**

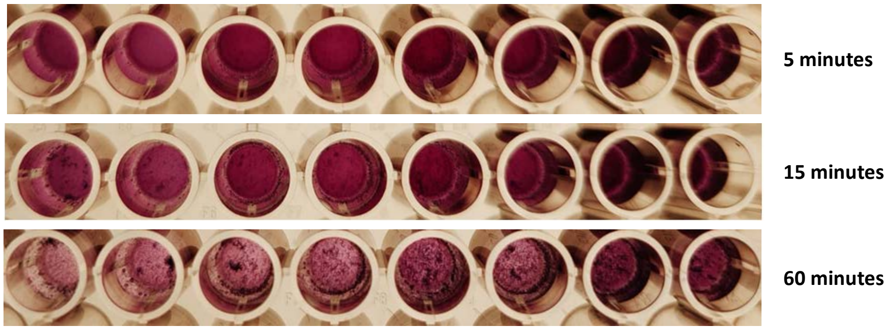

Suppl. Fig. S3. Time-dependent aggregation of formazan dissolved in ammonia-DMSO
Pure formazan was added at the quantities indicated below to duplicate wells containing 100 µl of PBS. Hundred µl of solvents were added and the 96-well plates were shaken at 200 rpm. Images were taken 5, 15, and 60 minutes after adding the solvent.
Quantity of formazan added per well: 12.5 µg - **wells 1 & 2**; 25.0 µg **- wells 3 & 4**; 37.5 µg – wells **5 & 6**; 50.0 µg - wells **7 & 8**.

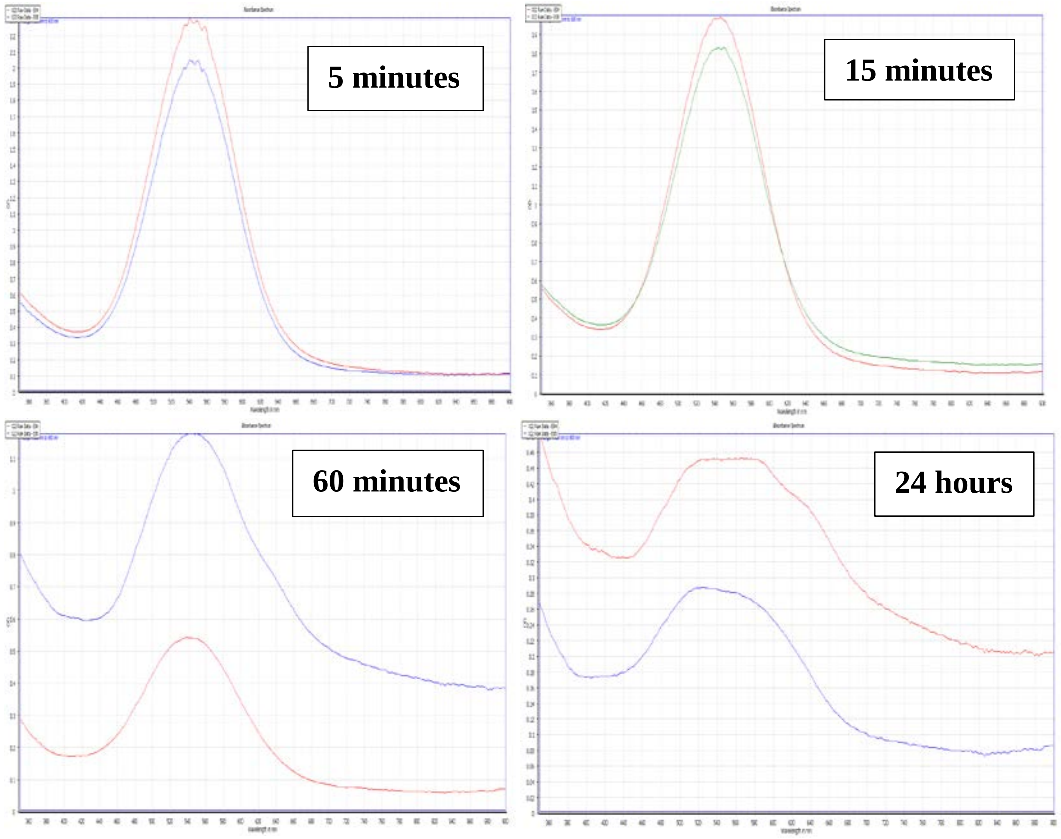

Suppl. Fig. S4. Time-dependent changes of the absorption spectrum of formazan dissolved in ammonia-DMSO
Spectra of duplicate wells were recorded 5, 15, 60 minutes, and 24 hours after adding ammonia-DMSO to wells containing 12.5 µg formazan in 100 µl PBS.

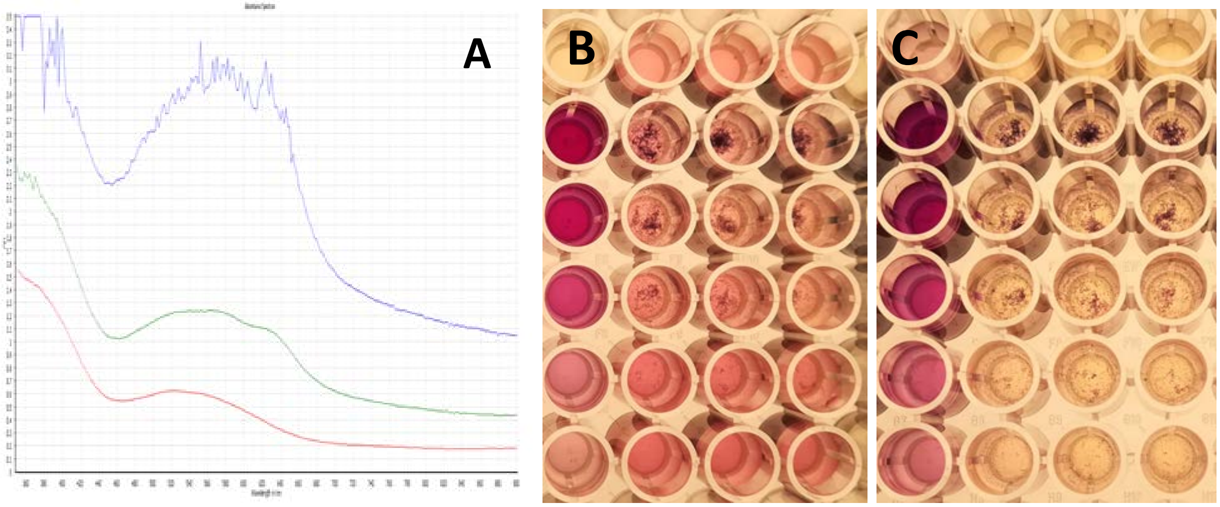

Suppl. Fig S5. Absorbance spectra of triplicate wells recorded 1 hour after the addition of ammonia-DMSO to wells containing 18x10^8 *E. coli* cells/ml subjected to the MTT assay (A). Images of a 96-well plate taken 1 hour (B) and 24 hours (C) after adding ammonia-DMSO. Wells contained medium (top row) or *E. coli* in the range 0.8x10^8 – 18x10^8 cells/ml. Ammonia-DMSO was added to solubilize formazan crystals after 30 min. of incubation with MTT solution.

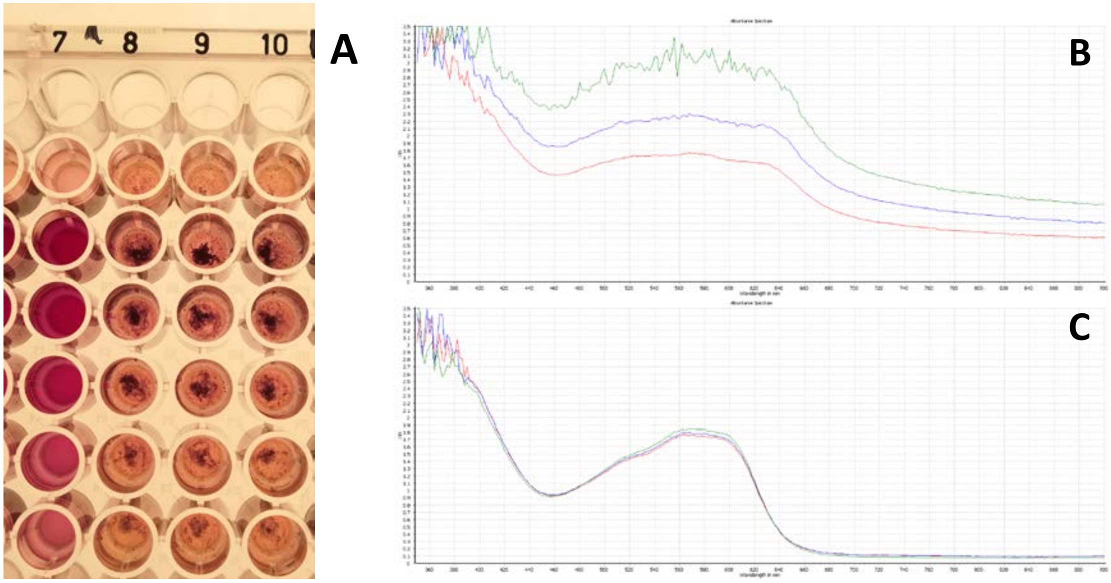

Suppl. Fig S6. Image of a 96-well plate (A) and absorbance spectra of triplicate wells B & C).
A) Wells containing 100 µl of BHI medium (top row) or 100 µl of *S. aureus* culture (1.5 – 57x10^8 cells/ml) were subjected to the MTT assay. Formazan was solubilized with ammonia-DMSO. The image was captured 2 hours after addition of the solvent.
B) Absorption spectra of triplicate wells containing 8x10^8 *S. aureus* cells/ml subjected to the MTT assay and solubilized with ammonia-DMSO. Spectra were recorded 2 hours after the addition of the solvent.

C) Absorption spectra of triplicate wells containing $8x10^8$ cells/ml, 2 hours after addition of 5% SDS-buffered DMSO. All other conditions were as in Panel B.

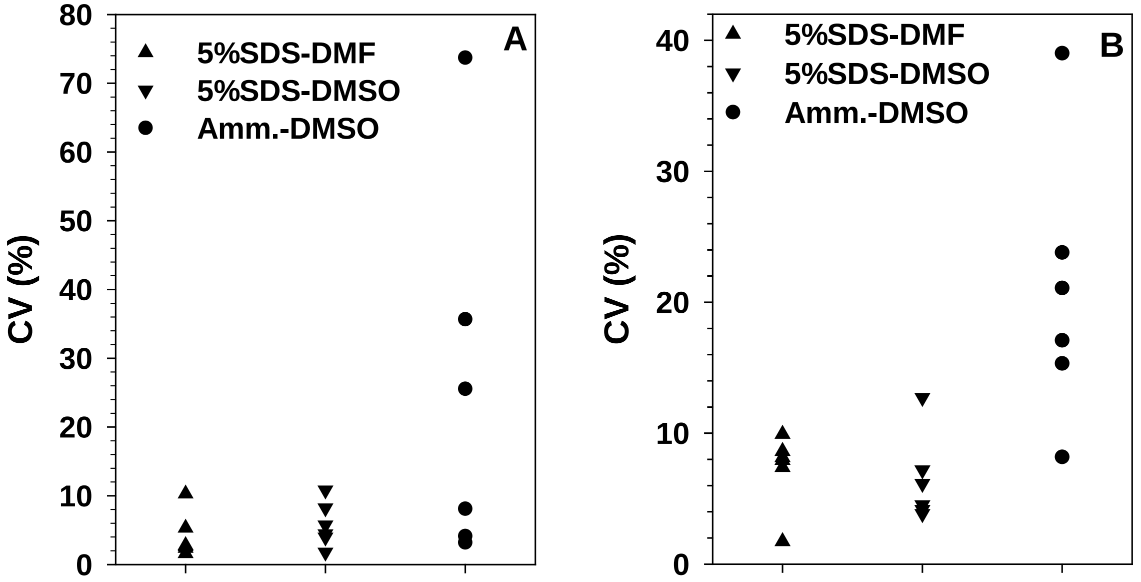

Suppl. Fig S7. Inter assay coefficient of variation (CV).
Panel A: *E. coli*; **Panel B:** *S. aureus*

E. coli	R^2	**p**
5% SDS-DMF	0.992	<0.001
5% SDS-DMSO	0.991	<0.001
Ammonia-DMSO	0.788	0.018
S. aureus **(up to 5.7x10^9 cells/ml)**		
5% SDS-DMF	0.769	0.022
5% SDS-DMSO	0.841	0.010
Ammonia-DMSO	0.288	0.272
S. aureus **(up to 5.7x10^9 cells/ml)**		
5% SDS-DMF	0.979	0.010
5% SDS-DMSO	0.990	0.005
Ammonia-DMSO	0.921	0.040

Suppl. Table 1. Coefficient of determination (R^2) and p values obtained by linear regression analysis for *E. coli* and *S. aureus* cultures subjected to the MTT assay and solubilized with the listed solvents.

Data were derived from Figs. 3 D and 4 D. For *S. aureus*, R^2 and p values were calculated for the entire range of cell densities (0 - 5.7x10^9 cells/ml) and for the linear part of the graph (0 – 8x10^8 cells/ml)
